# Supplementary material for: Silencing of circCRIM1 Drives IGF2BP1-Mediated NSCLC Immune Evasion
Source: Cells. 2023 Jan 10;12(2):273. doi: 10.3390/cells12020273 (PMC9856323; doi:10.3390/cells12020273)
Supplement: Supplementary file 1 [file cells-12-00273-s001.zip › cells-2062587-supplementary file.pdf]

Supplementary Information

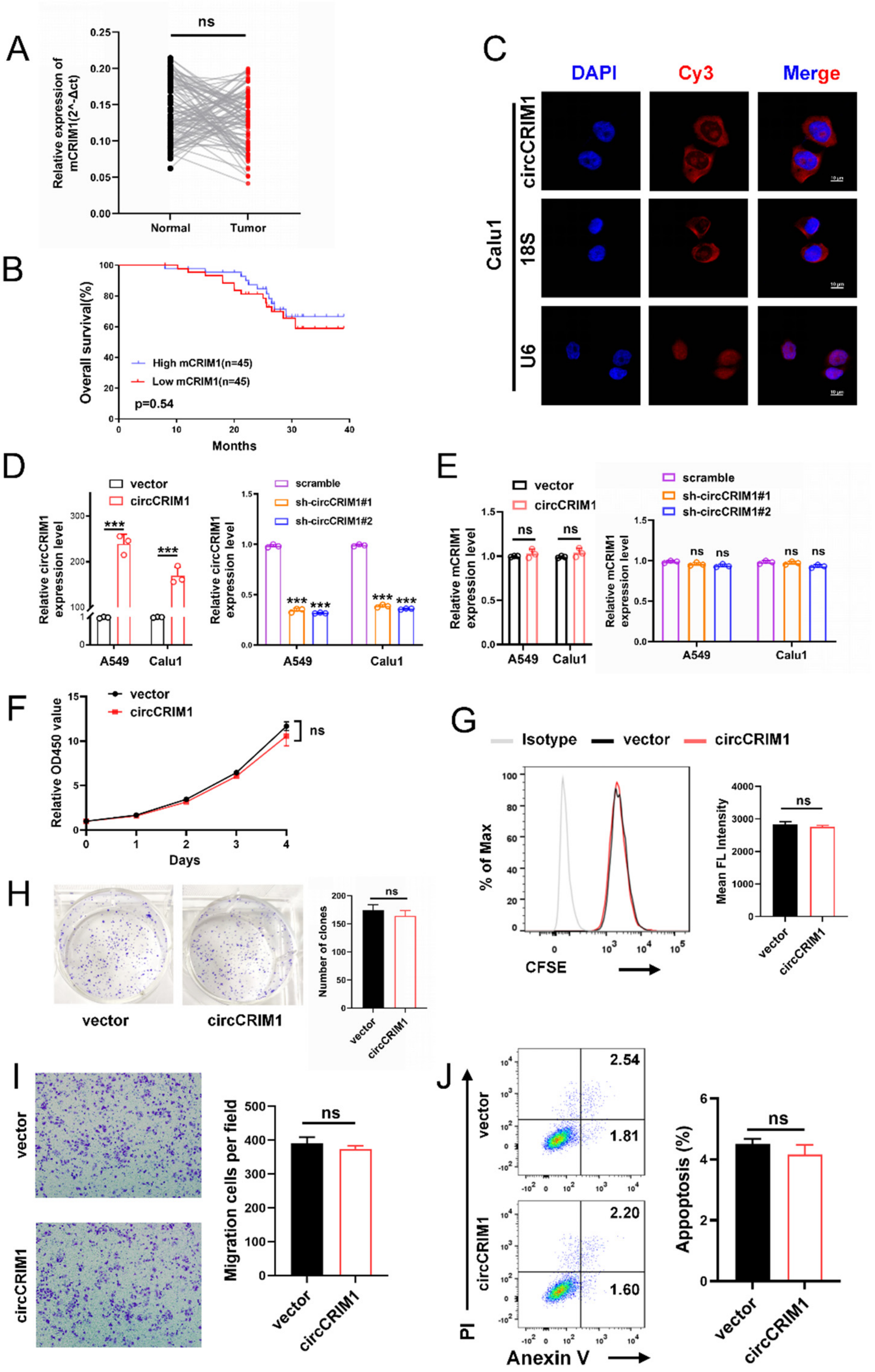

Figure S1. Identification and function of circCRIM1. (A) The expression of circCRIM1 was detected with qRT-PCR in 90 pairs of NSCLC and adjacent normal lung tissues, GAPDH was used as an internal control. Statistics analysis by paired t-test. (B) Kaplan-Meier curves of OS in NSCLC patients. Patients were grouped by the median mCRIM1 expression. P-value was

calculated using a log-rank test. (C) Identification of circCRIM1 cytoplasmic and nuclear distribution by FISH in Calu1 cells. (D-E) The expression of circCRIM1 and mCRIM1 were detected by qRT-PCR in A549 and Calu1 cells transfected with control, circCRIM1 or sh-circCRIM1 vectors. Statistics analysis by unpaired t-test or one-way ANOVA. (F-H) Cell proliferation were measured by the CCK-8 assay, colony formation and CFSE assay in the A549 cells transfected with control and circCRIM1 vectors. Statistics analysis by two-way ANOVA(F) or unpaired t-test(G,H) (I) Cell migration was evaluated by transwell assays of in the A549 cells transfected with control and circCRIM1 vectors. Statistics analysis by unpaired t-test. (J) Apoptosis assay was conducted in the A549 cells transfected with control and circCRIM1 vectors. Statistics analysis by unpaired t-test. Data shown as mean ± SD; ns, not significant; *$p < 0.05$, **$p < 0.01$, ***$p < 0.001$.

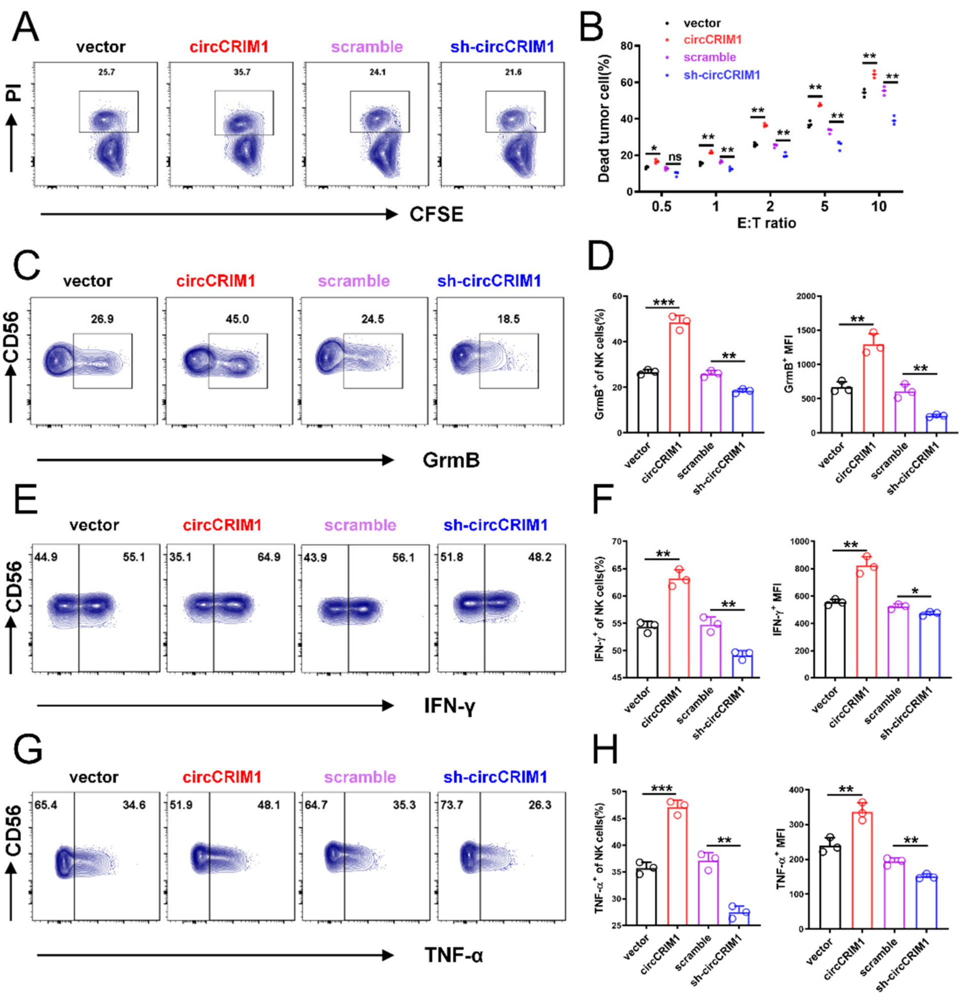

Figure S2. Overexpression of circCRIM1 inhibits immune evasion of lung cancer cells from the killing of NK cells (A) Flow cytometry analysis of dead cells in all CFSE labeled A549 cells that were stably transfected with vector, sh-circCRIM1#2 and circCRIM1 and co-cultured with activated NK cell for 24h. E:T ratio (effector to target cell ratio) =2:1.(B) Flow cytometry analysis displaying the percentage of dead tumor cells in co-culture assay with different E:T ratio. Statistics analysis by unpaired t test. (C-H) Flow cytometry analysis of Granzyme B, IFN-γ and TNF-α expression of NK cells co-cultured with A549 cells for 24h. E:T ratio =2:1. Statistical analysis by one-way ANOVA (D, F, H). Data shown as mean ± SD; ns, not significant; *$p < 0.05$, **$p < 0.01$, ***$p < 0.001$.

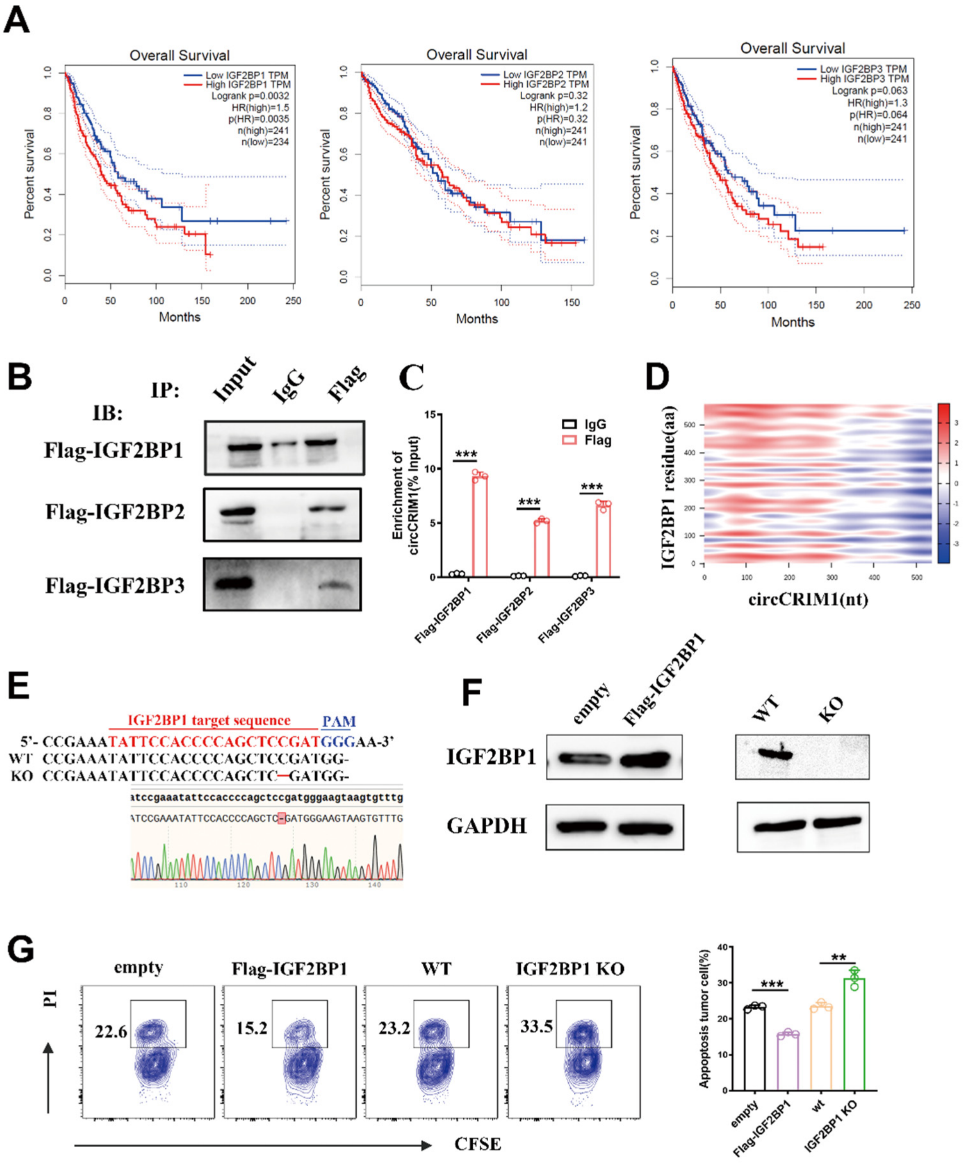

Figure S3. IGF2BP1 facilitates immune evasion in lung cancer. (A) Kaplan-Meier curves showing overall survival among patients with high IGF2BP1/2/3 versus low IGF2BP1/2/3 in patients of NSCLC from TCGA. (B) RNA immunoprecipitation (RIP) assays in Calu1 cells using IGF2BP1/2/3 and IgG antibody. (C) The flag-IGF2BP1/2/3-enriched circCRIM1 relative to the IgG-enriched value was calculated by qRT-PCR in Calu1 cells. (D) The heatmap of interactions between circCRIM1 and IGF2BP1 from catRAPID website. Statistics analysis by unpaired t test. (E) The knockout of IGF2BP1 was validated by sanger sequencing. (F) Western blot analysis was showed for relative expression of IGF2BP1 in A549cells IGF2BP1 knockout or overexpressed. GAPDH was used as a loading control. (G) Flow cytometry analysis was

showed for the apoptosis of IGF2BP1 knockout or overexpressed A549cells cocultured with pre-activated CD8+T cells for 24h. E:T ratio =2:1. Statistics analysis by unpaired t test. Data shown as mean ± SD; ns, not significant; *p < 0.05, **p < 0.01, ***p < 0.001.

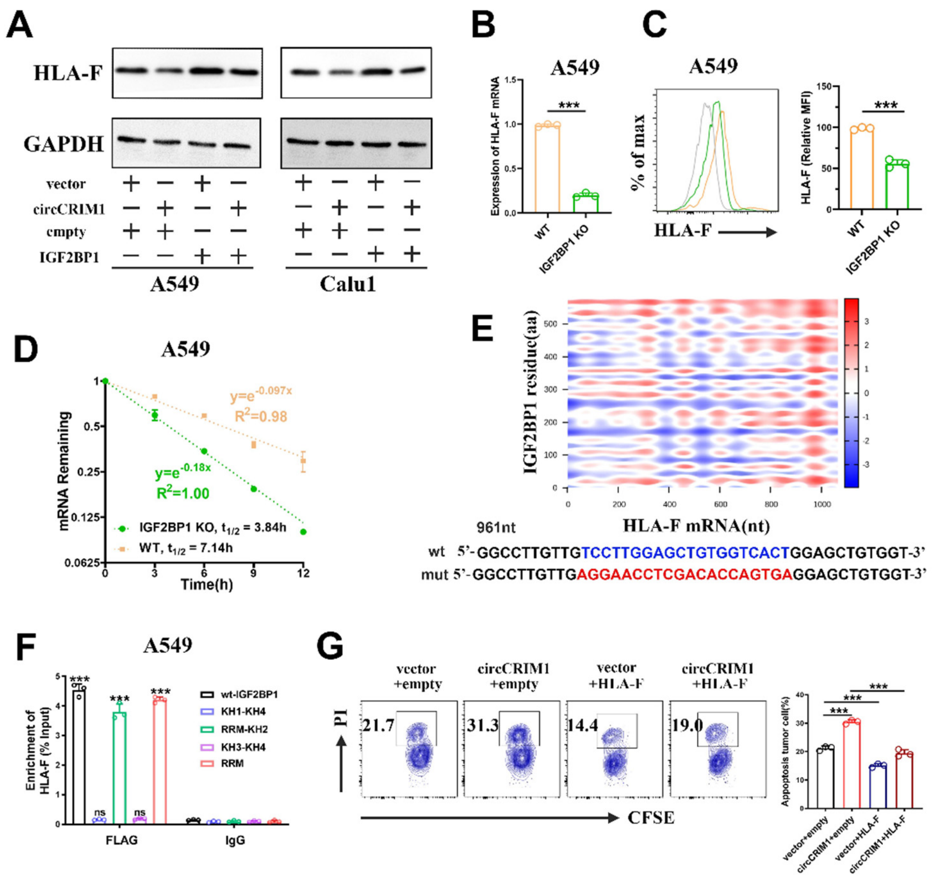

Figure S4. CircCRIM1 downregulates HLA-F expression via interacting with IGF2BP1 to facilitate immune evasion of NSCLC. (A) Western blot analysis was showed for relative expression of HLA-F in A549cells stably transfected with vector or circCRIM1, and co-transfected with IGF2BP1 or empty vector. (B) The expression of HLA-F mRNA was detected by qRT-PCR in wt and IGF2BP1 knockout A549 cells. (C) Flow cytometry analysis was showed for the expression of wt and IGF2BP1 knockout A549 cells. Statistics analysis by unpaired t test. (D) The relative RNA levels of HLA-F mRNA were analyzed by qRT-PCR and the mRNA half-life was calculated after treatment with actinomycin D at the indicated time points in wt and IGF2BP1 knockout A549 cells. (E) The heatmap of interactions between HLA-F mRNA and IGF2BP1 from catRAPID website, and the sequence of wt and mutant HLA-F mRNA. (F) RIP analysis for HLA-F mRNA enrichment in A549 cells transiently transfected with plasmids containing the indicated FLAG-tagged full-length or truncated constructs. Statistics analysis by unpaired t test. (G) Flow cytometry analysis was showed for the apoptosis of A549cells stably transfected with vector or circCRIM1, and co-transfected with HLA-F or empty vector, and cocultured with pre-activated CD8+T cells for 24h. E:T ratio =2:1. Statistics by one-way ANOVA with Tukey's multiple comparison analysis. Data shown as mean ± SD; ns, not significant; *p < 0.05, **p < 0.01, ***p < 0.001.

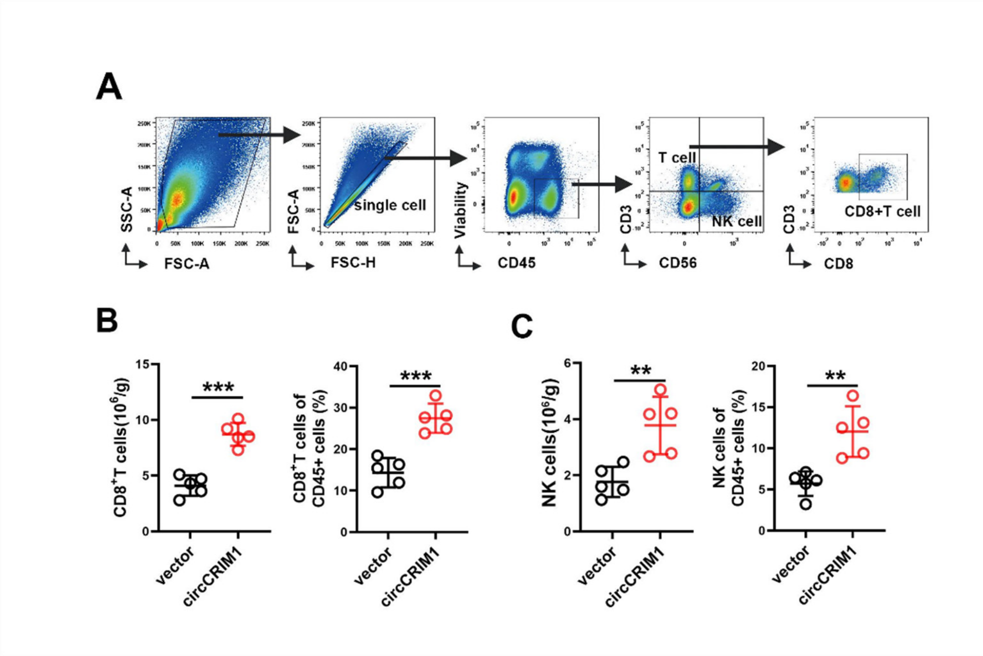

Figure S5. Overexpressed circCRIM1 facilitates the response of anti-tumor in mouse model. (A) Gating strategy for flow cytometry analysis in mouse model. (B) Representative flow cytometric analysis displaying the percentage of CD8+ T cell and NK cell in the tumor. Statistics analysis by unpaired t test. Data shown as mean ± SD; ns, not significant; *$p < 0.05$, **$p < 0.01$, ***$p < 0.001$.

Table S1. The sequences of primers or probes used for experiments in this study.

		**Primers for qPCR**
**Gene**	**Primer**	**Sequence (5′-3′)**
circCRIM1	Forward	TGTGGAATGCCCTCCTGTTCA
	Reverse	GGCACATATCCTGACTTGGAAACT
mCRIM1	Forward	AGCCGGGAAACCTGAACATACT
	Reverse	AACAGGAGGGCATTCCACAGT
GAPDH	Forward	CAATGACCCCTTCATTGACC
	Reverse	TTGATTTTGGAGGGATCTCG
GAPDH-divergent	Forward	TCAAGAAGGTGGTGAAGCAGGC
	Reverse	ATGCCAGTGAGCTTCCCGTT
U1	Forward	ACTTACCTGGCAGGGGAGATACC
	Reverse	CCACTACCACAAATTATGCAGTCG
U6	Forward	GCTTCGGCAGCACATATACTAAAAT
	Reverse	CGCTTCACGAATTTGCGTGTCAT
HLA-F	Forward	GCGCTTCTATGAGGCAGAGGAAT
	Reverse	GCGTCTCCTTCCCATTCTCCAA
		**Primers for plasmid construction**
**Vector**	**Primer**	**Sequence (5′-3′)**
IGF2BP1	Forward	GGAATTCATGAACAAGCTTTACATCGGCAACC
	Reverse	CCGCTCGAGCTTCCTCCGTGCCTGGGC
IGF2BP2	Forward	GGAATTCATGATGAACAAGCTTTACATCGGGAACC
	Reverse	CCGCTCGAGCTTGCTGCGCTGTGAGGC
IGF2BP3	Forward	GGAATTCATGAACAAACTGTATATCGGAAACCTC
	Reverse	CCGCTCGAGCTTCCGTCTTGACTGAGGTG
3xFlag IGF2BP1	Forward	GGAATTCATGAACAAGCTTTACATCGGCAACC
RRM	Reverse	CCGCTCGAGATCGGGGATGTAGGAGACCT
3xFlag IGF2BP1	Forward	CGGAATTCGACATCCCCCTTCGGCTCC
KH1-KH4	Reverse	CCGCTCGAGCTTCCTCCGTGCCTGGGC
3xFlag IGF2BP1	Forward	GGAATTCATGAACAAGCTTTACATCGGCAACC
RRM-KH2	Reverse	CCGCTCGAGTATTTCCTGCTCGGCCCTGCA
3xFlag IGF2BP1	Forward	CGGAATTCCAGGAGATGGTGCAGGTGT
KH3-KH4	Reverse	CCGCTCGAGCTTCCTCCGTGCCTGGGC
circCRIM1	Forward	CTAATGACTTTTTTTTTATACTTCAGATGAGAACTGGACTGATGA CCAACT

	Reverse	GCCTAATTCTTTTCCTTGCTTCTTACCTTGTTGGCAAAGTACAGC AACCAT
HLA-F	Forward	GGAATTCATGGCGCCCCGAAGCCTC
	Reverse	CCGCTCGAG CACTGCAGCCTGAGAGTAGCTCCCT
IGF2BP1 sgRNA1	Forward	CACCGTATTCCACCCCAGCTCCGAT
	Reverse	AAACATCGGAGCTGGGGTGGAATAC
IGF2BP1 sgRNA2	Forward	CACCGAGCGTGACCCCCGCGGACT
	Reverse	AAACAGTCCGCGGGGGTCACGCTC
IGF2BP1 sgRNA3	Forward	CACCGAGCACAAGATCTCCTACAG
	Reverse	AAACCTGTAGGAGATCTTGTGCTC
		**ShRNA oligo sequences**
**Vector**	**Oligonucleotide**	**Sequence (5′-3′)**
sh-circCRIM1#1	Forward	CCGGCAACAAGATGAGAACTGGACTCTCGAGAGTCCAGTTCTCA TCTTGTTGTTTTTG
	Reverse	AATTCAAAAACAACAAGATGAGAACTGGACTCTCGAGAGTCCA GTTCTCATCTTGTTG
sh-circCRIM1#2	Forward	CCGGTGCCAACAAGATGAGAACTGGACTCGAGTCCAGTTCTCAT CTTGTTGGCATTTTTG
	Reverse	AATTCAAAAATGCCAACAAGATGAGAACTGGACTCGAGTCCAGT TCTCATCTTGTTGGCA
scramble	Forward	CCGGCAACAAGATGAAGAGCACCAACTCGAGTTGGTGCTCTTCA TCTTGTTGTTTTTGGTACC
	Reverse	AATTGGTACCAAAAACAACAAGATGAAGAGCACCAACTCGAGT TGGTGCTCTTCATCTTGTTG
		**Oligonucleotides for FISH and RNA pull-down**
**Oligonucleotide**		**Sequence (5′-3′)**
circCRIM1 FISH		TTGGTCATCAGTCCAGTTCTCATCTTGTTGGCAAAGTACAGCAAC -Cy3
U6 FISH		Provided by Ribobio
18S FISH		Provided by Ribobio
Sense probe for circCRIM1 pull-down		GGTCATCAGTCCAGTTCTCATCTTGTTGGCAAAGTACAGC-Biotin
Anti-sense probe for circCRIM1 pull-down		GCTGTACTTTGCCAACAAGATGAGAACTGGACTGATGACC-Biotin
